# Tapered Optical Fiber Sensor for Label-Free Detection of Biomolecules

**DOI:** 10.3390/s110403780

**Published:** 2011-03-28

**Authors:** Ye Tian, Wenhui Wang, Nan Wu, Xiaotian Zou, Xingwei Wang

**Affiliations:** 1 Department of Electrical and Computer Engineering, University of Massachusetts Lowell, One University Ave., Lowell, MA 01854, USA; E-Mails: wwhclp02@gmail.com (W.W.); nan_wu@student.uml.edu (N.W.); 2 Biomedical Engineering and Biotechnology Doctoral Program, University of Massachusetts Lowell, One University Ave., Lowell, MA 01854, USA; E-Mail: xiaotian_zou@student.uml.edu

**Keywords:** optical fiber sensor, biosensor, label-free detection, evanescent wave, MEMS

## Abstract

This paper presents a fast, highly sensitive and low-cost tapered optical fiber biosensor that enables the label-free detection of biomolecules. The sensor takes advantage of the interference effect between the fiber’s first two propagation modes along the taper waist region. The biomolecules bonded on the taper surface were determined by demodulating the transmission spectrum phase shift. Because of the sharp spectrum fringe signals, as well as a relatively long biomolecule testing region, the sensor displayed a fast response and was highly sensitive. To better understand the influence of various biomolecules on the sensor, a numerical simulation that varied biolayer parameters such as thickness and refractive index was performed. The results showed that the spectrum fringe shift was obvious to be measured even when the biolayer was only nanometers thick. A microchannel chip was designed and fabricated for the protection of the sensor and biotesting. Microelectromechanical systems (MEMS) fabrication techniques were used to precisely control the profile and depth of the microchannel on the silicon chip with an accuracy of 2 μm. A tapered optical fiber biosensor was fabricated and evaluated with an Immune globulin G (IgG) antibody-antigen pair.

## 1. Introduction

Great efforts have been exerted in the development of optical fiber biosensors for the determination of various analytes, including DNA, proteins, antigens and cells. Those sensors are based on absorbance [1], reflectance [2], fluorescence [3], chemiluminescence [4], bioluminescence [5], or refractive index (RI) measurements [6]. Labels are necessary in most of them. However, the labels cause challenges in biomedical research due to the difficulty of finding an appropriate label that functions equivalently for all molecules [7], the negative effects on the biomolecules, the time-consuming pretreatment and high operation costs. Therefore, for many applications label-free methods are more attractive. Though some label-free biosensors based on surface plasmon resonance (SPR) [8], Fiber Bragg Gratings (FBG) [9], and various interferometers have been developed [10] and exhibit good performance, we intend to introduce a new choice featuring more advantages and potential, which is a simple tapered single-mode fiber biosensor. Its miniature size and mechanical flexibility allow convenient integration with current medical tools for sensing in inaccessible locations when using optical intensity demodulation, and multiple parameters of the tapered fiber profile can be optimized for different applications.

A tapered optical fiber sensor (TOFS) is an excellent candidate for sensing. Single-mode biconical tapered fibers can be applied as physical sensors for measurements of temperature, displacement, and refractive index [11]. They can also be used to couple light into a micro-cavity sensor [12], to be further fabricated as a nanoresonator sensor [13]. A fiber Mach–Zehnder interferometer realized by concatenating two single-mode tapered fibers can also be used as a refractive index sensor [14]. Due to their small size, the aforementioned tapered fibers are attractive for a variety of applications. However, their applications as biosensors are still being developed.

To diversify the applications, more complex tapered fiber structures can be developed. A refractometer can be realized by writing a single long-period grating (LPG) into a biconical tapered fiber [15] or a pair of LPGs into the two sides of the taper region [16]. Some FBG based tapered fibers can measure fluid levels [17]. However, the complex tapered fibers with LPG or FBG still suffer from high fabrication costs. TOFS also have been used for biosensing applications such as the detection of pathogens, protein or cell concentration, and DNA hybridization. Thus far most of TOFS utilize fluorescent labels [18] or SPR [19] to amplify the signal associated with the presence of the bio-target. However, the disadvantages of either a labeling step or additional complexity are undesirable. In this paper, we designed and fabricated a simple tapered single-mode fiber structure to realize a low cost optical fiber biosensor featuring a miniature sensing probe, label-free direct detection, and high sensitivity.

## 2. Principle

**Figure 1.**
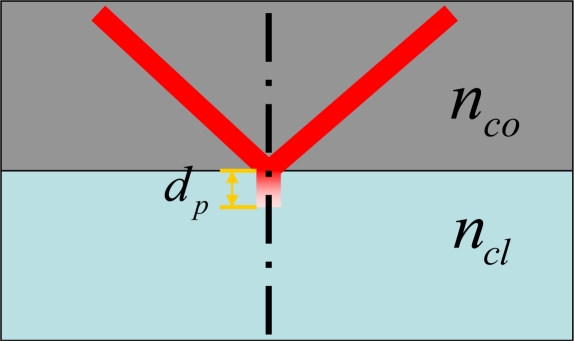
Penetration depth. The evanescent field decays to 1/e of its value at the core-cladding interface.

Optical fiber usually consists of a core and a cladding with different refractive indices. When light propagates, the main guided field is defined in the core, and the evanescent field is exponentially decayed in the cladding. After a distance of extension, the evanescent field decays to 1/e of its value at the core-cladding interface. As shown in Figure 1, this distance is defined as penetration depth, and it is mathematically described as:

(1) $$d_{p}=\frac{\lambda }{2\pi \sqrt{n_{\mathrm{co}}^{2}\;{\text{sin}}^{2}\theta -n_{\mathrm{cl}}^{2}}}$$

where *λ* is the wavelength of the light source, *θ* is the angle of incidence of the light at the core/cladding interface, *n_co_* and *n_cl_* are the refractive indices of the core and the cladding, respectively.

Optical fibers were originally designed for low loss communication, so the penetration depth is far smaller than the cladding thickness and there is almost no interaction between the optical field and the surrounding environment. For some sensing applications, the evanescent field needs to be exposed to the environment. Tapering fiber is a good solution to make the interaction between the evanescent wave and the surrounding target possible.

**Figure 2.**
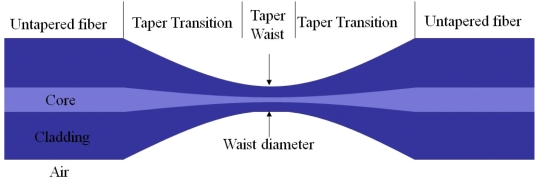
Schematic of a tapered fiber. The tapered fiber consists of three contiguous parts: one taper waist segment with small and uniform diameter, and two conical transition regions with gradually changed diameter. The ends of conical transition regions are untapered fibers.

Tapered fiber is normally made by pulling the optical fiber when it is heated to its softening temperature to reduce the diameter to tens of micrometers. As a result, the tapered fiber consists of three contiguous parts: one taper waist segment with small and uniform diameter, and two conical transition regions with gradually changed diameter. The ends of conical transition regions are untapered fibers. A typical tapered fiber is shown in Figure 2. In reality, the fiber core material in the waist and transition regions do not have clear boundaries with the cladding material due to their mixing after heating.

In the untapered single mode fiber, the thin core is surrounded by the cladding with a lower refractive index. All the light is guided within the core due to total internal reflection. Light propagation through an optical fiber can be described by wave theory. The properties of light in the fiber core are determined by the number of modes which are directly related to V number, given as:

(2) $$V=\frac{2\pi }{\lambda }r{\left(n_{\mathrm{co}}^{2}-n_{\mathrm{cl}}^{2}\right)}^{1/2}$$

where *r* is the radius of the core. The only transverse mode of light in the core of untapered single mode fiber is the fundamental mode HE_11_.

However, at the transition region, along with the decreased diameter, the core of the fiber almost gets mixed together with the surrounding cladding to form a medium whose refractive index is very close to that of the cladding. This medium can be taken as an air-cladding core, which has a larger radius than that of the original single mode fiber core in the most part of the region, and a larger numerical aperture due to larger refractive index difference between the cladding and the air. According to Equation (2), this region functions like a multimode fiber that supports multiple modes. This transition region is normally divided into two distinct categories: adiabatic and nonadiabatic. In the condition of adiabatic transition, the fundamental fiber mode HE_11_ can be carried out with efficiency as high as 99.5% and the contribution of higher order modes is insignificant and is not taken into consideration [19]. In the condition of nonadiabatic transition, some high-order modes can be excited, but the first two modes HE_11_ and HE_12_ are significant if the V number is controlled properly.

In the taper waist region, part of the light energy is not confined by the thin waist, around which an evanescent field is generated, and primarily the first two modes HE_11_ and HE_12_ coupled from one end of nonadiabatic transition region propagate at the air-cladding interface. After the interference, HE_11_ and HE_12_ modes couple back into fundamental mode at the other end of nonadiabatic transition region. The second coupling efficiency depends on relative phase Δ∅ = Δ*k*·*l*, where Δ*k* is relative propagation constant and *l* is coupling distance. Δ*k* can be tuned by changing the environment medium. *l* can be changed by taper waist length. The interference effect between HE_11_ and HE_12_ along the waist region incurs fringes in transmission spectrum, typically a sinusoidal behavior [20]. If Δ*k* or *l* changes, the spectral response of the taper will shift correspondingly [11]. Such interference and spectral response shifts are used for the biosensing in this paper. In order to ensure this interference, the taper waist diameter needs good control. If it is less than one micrometer approximately the interference effect does not exist for 1,550 nm light, because at such size the waist has similar properties as the core in the single mode fiber and it no longer supports HE_12_ mode. This diameter limit can be calculated by substituting the single mode V number limit (2.405), and the refractive indices of cladding and air into Equation (2). Therefore, we normally control the taper waist to be about ten micrometers in diameter.

## 3. Fabrication

### 3.1. Tapered Sensor Fabrication

**Figure 3.**
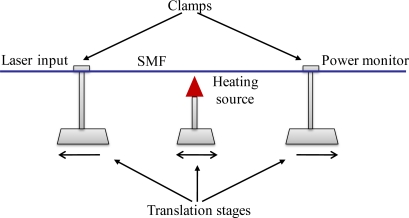
A typical fabrication setup. Two clamps were used to fix the SMF onto two translation stages. A torch flame or a CO_2_ laser could be utilized as heating source. A CTS excited one end of the fiber and monitored the spectrum response from the other side.

The parameters such as taper waist diameter, length, and transition shape, are critical for a specially required interference spectrum. By controlling the fabrication conditions such as pulling speed, heating length and temperature, tapered fiber with different shapes and properties can be fabricated. Figure 3 becomes a typical fabrication setup. Single-mode fibers (SMF) with core/cladding diameters of 8/125 μm were used. Its protective coating was removed over a section of about 15 mm long for heating. Two clamps were used to fix the SMF onto two translation stages (NRT 100, Thorlabs). A torch flame or CO_2_ laser was used as a heating source. A laser in a component test system (CTS) (Si 720, Micron Optics) excited one end of the fiber. The spectrum response from the other side of the fiber was monitored.

The fiber could be pulled from one end [11] or both ends [19], and the heating source could be fixed [14] or oscillated [16]. In order to prevent too many variable factors that may incur uncertainties in the optical performance of the tapered fiber, we only pulled one end. For the heating source, because a torch flame could not provide repeatable heating size and temperature, an Engraving Systems (Zing-Model 10000) CO_2_ laser was applied. Its laser source was digitally controlled and permanently aligned. Its adjustable laser power, speed, and pulse frequency provided much heating flexibility. In this case, we applied an oscillation heating with frequency 2 Hz within a 5 mm fiber section.

**Figure 4.**
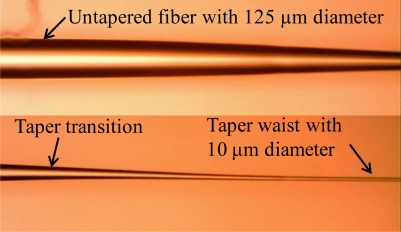
A typical fabricated tapered fiber. The untapered fiber was 125 μm of diameter, and the taper waist was about 10 μm of diameter. The taper transition region had a gradually changed diameter.

During the fabrication process, the fiber must be held straight all the time. After one second of heating startup, the translation stage was started with acceleration of 0.3 mm/s^2^ and constant speed of 0.9 mm/s to move a defined length. Transmission spectrum data was collected by a spectrum response recording system for further analysis. Figure 4 shows a typical fabricated fiber.

Tapered fiber could also be fabricated by a special programmable fusion splicer with discharge electrode [21] or filament. An optical fusion splicer (S177A) was used for fabrication. This method was automatic, but the tapered length was limited, which could reduce the biosensing sensitivity.

### 3.2. Microchannel Holder Fabrication

**Figure 5.**
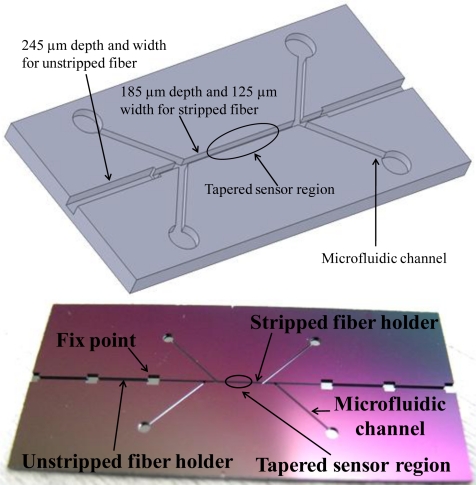
**(a)** (upper) Chip structure. Both ends of the channel were in 245 μm depth and width so they could hold the unstripped sections of the fiber, while the middle of the channel was in 185 μm depth and 125 μm width to hold the stripped fiber. The four microfluidic channels branches were designed for inlets and outlets of bio-target and fluid, respectively. **(b)** (lower) Real etched chip.

The tapered fiber sensor was thin and fragile after the fabrication. A microchannel chip was designed and fabricated for the sensor protection and further biotesting. The schematic of the microchannels on a silicon chip was shown in Figure 5(a).

Both ends of the channel were in 245 μm depth and width so they could hold the unstripped sections of the fiber, while the middle of the channel was in 185 μm depth and 125 μm width to hold the stripped fiber. The four microfluidic channels branches were designed as inlets and outlets for the bio-target and fluid, respectively. Microelectromechanical systems (MEMS) fabrication techniques, specifically photolithography and deep dry etching, were used to precisely control the profile and depth of the microchannel on the silicon chip with an accuracy of 2 μm. First, a mask with the desired profile of microchannels and microfluidic system was fabricated, and the mask pattern was transferred to a silicon wafer by a layer of photoresist. Then, the patterned wafer was etched with an inductively coupled plasma deep etcher. Figure 5(b) showed a real etched chip. The channel sizes were matched to fit the fiber dimensions. After the sensor was fixed into the microchannel chip, the chip surface was sealed by a layer of polydimethylsiloxane (PDMS) leaving the inlets and outlets open.

## 4. Results and Discussion

### 4.1. Experiments

**Figure 6.**
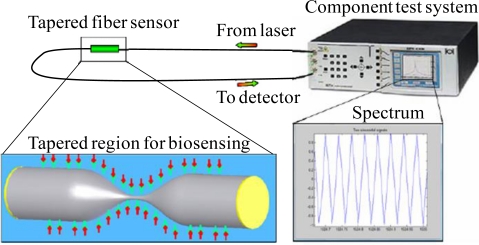
The CTS was used to excite laser into one end of the tapered fiber sensor, and monitor the transmission spectrum from the other end. The bio-target bonding in the tapered region could be analyzed through phase shift of the spectrum.

The biotest was set up as shown in Figure 6. The component test system was used to excite laser into one end of the tapered fiber sensor, and monitor the transmission spectrum from the other end. The bio-target bonding in the tapered region could be analyzed through the phase shifts of the spectrum.

For a typical test, IgG antibody-antigen pair was used to evaluate the performance of the tapered fiber biosensor. The biotest procedure was as follows: the tapered fiber was washed with deionized water and dried in air or by nitrogen gas; The 2% aminopropyltriethoxysilane solution in acetone was prepared for silanization; the prepared solution was injected into the sensing region for 30 minutes at room temperature; the resulting alkylaminosilane-derivatized tapered surface was cleaned by deionized water; a solution of 10% glutaraldehyde in 50 mM sodium phosphate-buffered saline (PBS) at pH 7.4 was injected onto the alkylaminosilane-derivatized surface for 2 hours to activate the silanized substrate, followed by washing out the excess of glutaraldehyde thoroughly with deionized water and drying; IgG antibody solution was injected onto the activated surface and kept for 2 hours at 4 °C, followed by washing out the physically adsorbed IgG antibody with deionized water and drying; The initial transmission spectrum of antibody layer was recorded by the CTS system; IgG anitigen solution was injected to the oriented antibody layer and kept for 2 hours at 4 °C, followed by washing out the remaining nonimmobilized antigen with deionized water and drying; The transmission spectrum of IgG antibody-antigen pair layer was recorded for comparison.

**Figure 7.**
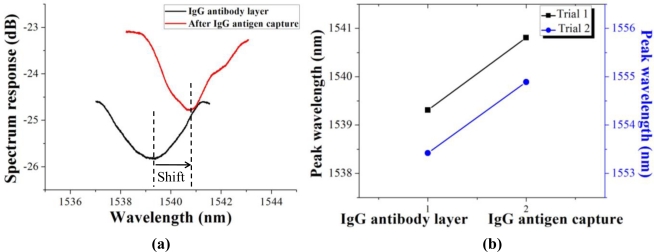
**(a)** The comparison between the initial transmission spectrum (black) and IgG antibody-antigen pair layer (red). An obvious redshift of 1.50 nm after the capture of the IgG antigen was monitored. **(b)** The captures of IgG antigen were successfully detected by demodulating the peak wavelength redshift of 1.50 nm and 1.47 nm, respectively in two trials.

The comparison between the initial transmission spectrum (black) and IgG antibody-antigen pair layer (red) was shown in Figure 7(a). There was an obvious redshift of 1.50 nm after the capture of the IgG antigen. The reason of the small changes of profile and intensity in spectrum response will be analyzed after a comparison with the simulation results. As the repeatability test results shown in Figure 7(b), the captures of IgG antigen were successfully detected by demodulating the peak wavelength redshift of 1.50 nm and 1.47 nm, respectively, in two trials.

### 4.2. Simulations

To better understand the influence of various biomolecules on the sensor, a numerical simulation that varied the thickness and refractive index of the bio-layer was performed. According to the dimensions and properties of the tapered fiber, a three-dimensional (3D) tapered fiber model was built to simulate the transmission spectrum response based on the Beam Propagation Method (BPM).

**Figure 8.**
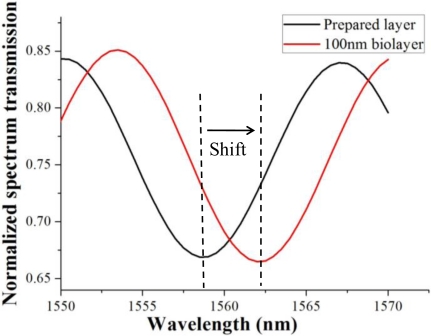
The transmission spectrum fringe redshift. The black curve indicated the transmission spectrum under a bio-test preparation layer with refractive index 1.33 and thickness 100 nm. After a target biolayer with refractive index 1.33 and thickness 100 nm was bonded above the preparation layer, the redshift 3.5 nm was represented by the red curve.

In the simulation model, taper waist length was 12 mm, transition lengths were 4 mm and 8 mm, and transition profile was exponential or conical. Untapered fiber diameter was 125 μm, and taper waist diameter was 10 μm. Background refractive index *n_b_* = 1, cladding refractive index *n_cl_* = 1.4629, core refractive index *n_co_* = 1.4682. The transmission spectrum fringe redshift when a layer with refractive index 1.33 was coated on the taper waist surface is shown in Figure 8. The black curve indicated the transmission spectrum under a bio-test preparation layer with refractive index 1.33 and thickness 100 nm. After a target biolayer with refractive index 1.33 and thickness 100 nm was bonded upon the preparation layer, the fringe shifted 3.5 nm to the red curve.

**Figure 9.**
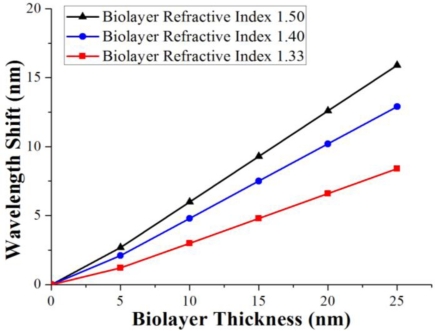
The phase shift with different biolayers thickness in nanometer level. Each of the three conditions has a linear phase shift response to the bonded biolayer thickness.

In general, the bonded biolayer should be in the nanometer range. To illustrate the potential to detect a very thin bio-layer, an optimized tapered fiber model with thinner and longer waist was used to calculate the influence of the presence of a thin biolayer at the taper waist. Figure 9 showed the phase shift with different biolayers’ thicknesses at the nanometer level. The conditions of three typical refractive indexes were given. Each of the three conditions has a linear wavelength shift response to the bonded biolayer thickness.

### 4.3. Analysis

The computer simulation showed perfect sinusoid spectrum profiles and redshifts after the bonding of biomolecule layers. However, the experimental results showed small changes in profile and intensity besides the obvious redshift. There might be some reasons for this. First, the tested tapered fiber sensor was not as ideal as the simulation model with the uniform taper waist and exponential taper transitions, so the spectrum was not a regular sinusoid due to more high-order propagation modes. Second, the biomolecule layers were not only bonded on the taper waist, but also the taper transitions which might change the propagation coefficient of the waveguide. Third, there might be physically adsorbed antigen which also changed the properties of the tapered waveguide. Fourth, a small intensity change was demonstrated in the simulation results (Figure 8), but the redshifts were close to linear (Figure 9), so the biosensing could be quantified from the linear redshifts, and the small intensity change in the experiment could be ignored. In all, a better experimental result should be obtained by further improved fabrication of the tapered fiber sensor and the more precise control of the biotesting.

## 5. Conclusions and Future Work

A low cost tapered fiber biosensor featuring a miniature sensing structure, label-free direct detection, and high sensitivity was presented. A miniature biosensor less than ten micrometers wide and several millimeters long was fabricated. Its high sensitivity was realized by the sharp spectrum fringe and sufficiently long biomolecule reaction region. A microchannel chip was designed and fabricated for the sensor’s protection and biotesting. Finally, the good performance of the fabricated biosensor was evaluated with an IgG antibody-antigen pair, and the influence of the biolayer on the tapered fiber was simulated to consolidate the theoretical analysis and experimental results. To minimize the whole system and lower the cost, an intensity interrogator is under study.
